# Correlation of the BI‐RADS assessment categories of Papua New Guinean women with mammographic parenchymal patterns, age and diagnosis

**DOI:** 10.1002/jmrs.422

**Published:** 2020-09-16

**Authors:** Ruth Pape, Kelly Maree Spuur, Jenny Maree Wilkinson, Pius Umo

**Affiliations:** ^1^ School of Medicine and Health Sciences Discipline of Medical Imaging Science University of Papua New Guinea Boroko NCD Papua New Guinea; ^2^ Pacific International Hospital Boroko NCD Papua New Guinea; ^3^ Faculty of Science School of Dentistry & Health Sciences Charles Sturt University Wagga Wagga NSW Australia

**Keywords:** BI‐RADS, breast density, breast pathology, mammographic parenchymal patterns, Papua New Guinea

## Abstract

**Introduction:**

Women with increased breast density are at increased risk of breast cancer. The aim of this research is to evidence for the first time the mammographic breast findings of Papua New Guinean (PNG) women and the relationship between Breast Imaging‐Reporting and Data System (BI‐RADS) assessment, mammographic parenchymal patterns (MPPs) and age.

**Methods:**

A retrospective analysis of 1357 mammograms of women imaged at the Pacific International Hospital (PIH) from August 2006 to July 2010 was undertaken. Mammographic findings were categorised using the BI‐RADS Atlas® 5th Edition. MPPs were recorded for each woman using the Tabár Pattern I‐V classification system. Age was recorded in years. Statistical analysis was by descriptive analysis and Kruskal–Wallis with Dunn’s post‐test and Spearman’s rho correlation for inferential analysis.

**Results:**

True pathological findings (benign and malignant); BI‐RADS 2–5 were noted in 111 women (8.2%); 1242 (91.5%) were negative. BI‐RADS categories for malignancy were reported in 16 (88.9%) of women aged 30 to 60 years. The lower risk Tabár Type I, II and III MPPs were associated with 94.4% (*n* = 17) of malignancies. Linear correlations between variables were weak and not statistically significant: age and Tabár pattern *r* = 0.031, *P* = 0.0261; age and BI‐RADS *r* = 0.018, *P* = 0.517; Tabár pattern and BI‐RADS *r* = 0.020, *P* = 0.459 (*n* = 1357).

**Conclusion:**

There was no correlation demonstrated between BI‐RADS category, age and MPP. Importantly, there was no correlation demonstrated between BI‐RADS categories 4 and 5 for breast malignancy and high‐risk Tabár Type IV and V MPPs. The results of this study again reflect that the incidence of breast cancer in PNG cannot be explained by breast density and suggest that any formalised screening program in PNG has a target age group aimed at women younger than that of Western screening programs.

## Graphical abstract

Women with increased breast density are at increased risk of breast cancer. The results of this study again reflect that the incidence of breast cancer in Papua New Guinea cannot be explained by breast density. There was no correlation demonstrated between BI‐RADS categories 4 and 5 for breast malignancy and high‐risk Tabár Type IV and V mammographic parenchymal patterns. The lower risk Tabár Type I, II and III mammographic parenchymal patterns were associated with 94.4% (*n* = 17) of malignancies.

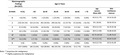

## Introduction

### Breast cancer

Breast cancer is the leading causes of mortality and morbidity amongst females worldwide.^1^ In 2018, more than 2.1 million new cases of breast cancer were diagnosed, accounting for one in four female cancer cases.^1^ Unlike Western countries, in Papua New Guinea (PNG) the age‐specific breast cancer incidence is documented as greatest in women aged between 35 and 54 years, with 75% of breast cancers being diagnosed in premenopausal women and falling incidence in post‐menopausal women.^2^ The previously reported peak incidence of breast cancer in PNG is in the 45 to 54 year old age group, 83.9% <54 years, 55.7% <45 years and 15.7% <35 years.^2^ The age‐standardised incidence figures, however, remain lower at 45.8/100,000 for PNG women, compared to that of Western countries including Australia, at 94.5/100,000.^3^

It is important to note that the true size of the PNG female population is not actually known and not all groups within PNG have equal access to health care if at all, and hence may not be accurately represented in the known data.^4^ Additionally, inadequate imaging services and limited education about breast cancer combined with cultural issues, including the use of traditional healers, mean that not all breast cancer cases will be documented as women do not necessarily present to the healthcare system for diagnosis and treatment.^2^ Further, women in PNG typically present with more advanced breast cancers (i.e. Stage III or Stage IV disease).^2^, ^4^, ^5^, ^6^ Histologically invasive ductal carcinomas are reported as the most common finding.^4^

Mammography is the most commonly used imaging tool for identifying breast pathology, including breast cancer.^7^ Reduction of breast cancer morbidity and mortality (13–28%)^8^, ^9^ has been evidenced worldwide through the introduction of mammography screening services, such as BreastScreen Australia (BSA), as recommended by global health organisations including the World Health Organisation (WHO).^10^

However, the WHO also recognises that mammography screening is not cost effective in a limited resource settings such as PNG which has logistical and geographical challenges along with a weak health system.^10^, ^11^

### Mammography in PNG

Currently, there is no BSA equivalent, in place in PNG.^11^, ^12^ There are only two screening type services in the capital Port Moresby. One is located in the main public hospital, Port Moresby General Hospital (PMGH) and is funded by the government. The other service is located in a private hospital, the Pacific International Hospital (PIH) where a free screening type program was established in 2005, sponsored by the PNG Motor Vehicle Insurance Limited (MVIL).

Hence, although the mammography provided at PIH is referred to in PNG as screening mammography, it is better aligned to the purpose of harm minimisation as described by the WHO, early diagnosis based on awareness of early signs and symptoms rather than asymptomatic screening.^10^

### Breast pathology reporting

Approaches to the reporting of mammographic images are known to vary between screening and diagnostic services and between countries worldwide. However, fundamental radiological interpretation of mammographic images includes the absence or presence of breast pathology and the amount of breast density present.

There are two common classification systems in use for classifying mammographic lesions, and these include the Tabár/Royal Australian and New Zealand College of Radiologists (RANZCR) classification, used by BreastScreen Australia and BreastScreen Aotearoa^13^ and in North America and most European countries^14^ the American College of Radiology (ACR) Breast Imaging‐Reporting and Data System (BI‐RADS).^15^

The aim of BI‐RADS is to standardise breast imaging reporting including mammography, ultrasound and magnetic resonance imaging (MRI), through a recommended reporting structure.^16^ The ACR BI‐RADS® Atlas Fifth Edition includes seven assessment categories, and three subcategories that report image evaluation, management recommendations and suspicion for malignancy.^15^ The ACR BI‐RADS assessment tool is routinely used for mammographic reporting at the PIH PNG.

### Breast density

A strong independent risk factor for breast cancer is breast density.^17^, ^18^ Breast density is defined as the relative amounts of radiopaque parenchymal tissue (dense) to radiolucent adipose (fatty) tissue as seen on a mammogram.^19^ The associated independent risk factor for breast density and breast cancer has been reported as being increased four to six times for women with a high ratio (dense) compared to low ratio (fatty), glandular to adipose tissue.^18^

The literature describes a number of longstanding classification systems that categorise and report breast density and the association between increased breast density and breast cancer risk including the BI‐RADS and Tabár systems.^15^, ^20^ The Tabár classification system has been chosen for use in this study as the population has been previously investigated using this system.^21^ The Tabár classification system categorises five types of parenchymal patterns: Patterns I, II and III are associated with a low risk for malignancy while Patterns IV and V have a high risk. The importance of breast density and its relationship to increased breast cancer risk^18^ and a reduction of sensitivity of mammography^22^ are evidenced by mammographic breast density (MBD) notification laws in the United States mandating the reporting of mammographic breast density in 32 states.^22^ Insurance cover in six of the abovementioned states is also mandated for supplemental screening.^23^ Mammography providers in PNG do not routinely report on breast density or notify women of their individual breast density, nor is supplemental screening (MRI, ultrasound or tomosynthesis) routinely encouraged.

### Age

In most Western countries, the target age for mammographic screening commences at 50 years and ends at 74 years.^24^ The relationship between reproductive age and breast cancer incidence is well established with exposure to oestrogen one of the key risk factors for breast cancer. Prolonged endogenous oestrogen exposure related to early menarche, decreased or null parity, first pregnancies after the age of 30 years, limited or no breastfeeding and late onset of menopause explain the relationship between the increased odds of breast cancer and increasing age.^25^ Women aged 40–49 years report increased tumour growth rates^26^ and those under 35 years are more likely to have higher grade, more poorly differentiated tumours with increased vascular invasion compared to older women.^27^, ^28^

Despite the plethora of published information on mammography, breast density and breast cancer, there is very little written about breast cancer in PNG with the few available papers concerning breast cancer dated between 1963 and 2004^2^, ^4^, ^5^, ^6^, ^27^, ^28^, ^29^ and more recently by the current authors between 2017 and 2019.^12^, ^21^, ^30^ This research aims to evidence the mammographic breast findings of the women of PNG and explore for the first time, relationships between BI‐RADS assessment, MPPs and age.

## Materials and Methods

A retrospective analysis of 1357 mammograms of women who had undergone imaging at the PIH from August 2006 to July 2010 was undertaken. These images are a subset of those taken during this period and simply represent the available images. Mammographic findings were categorised using the BI‐RADS Atlas® Fifth Edition lexicon,^15^ by an experienced Radiologist with 12 years of experience in breast image reporting who was unaware of any histopathological diagnosis. Women who had undergone mastectomy or lumpectomy were excluded from the study.

The mammographic images used for this study were previously retrospectively assigned one of five Tabár MPP categories by direct observation, and reported by Pape, Spuur and Umo (2017).^21^ Patient age was elicited from the data embedded in the images and recorded in years. Only mammograms of women who had indicated consent for their images to be used for research purposes were used for the study.

BI‐RADS scores were additionally correlated to formal diagnosis. Symptomatic presentations were also recorded.

### Ethics

Ethics approval and permission to collect data was granted through the University of Papua New Guinea, School of Medicine and Health Science Research Ethics Committee (Project Number 0118) and by the Medical Director and Chief Operating Officer of PIH. Consent to collaborate in this research project was granted from the Head of Division of Radiology at PIH.

### Statistical analysis

Descriptive analysis of the data was undertaken and following normality testing non‐parametric tests (Kruskal–Wallis with Dunn’s post‐test and Spearman’s rho correlation) were used for subsequent inferential analysis. BI‐RADS groups 0 and 4 were excluded from inferential analyses due to small sample size. Statistical analysis was completed using SPSS (IBM Corp. Released 2017. IBM SPSS Statistics for Windows, version 25.0. Armonk, NY: IBM Corp.); p < 0.05 was deemed to be statistically significant. Data distributions displayed in a violin plot were created using BoxPlotR.^31^

## Results

A total of 1357 mammograms were initially assessed. Women were aged between 20 and 80 years (Table 1). True pathological findings (benign and malignant); BI‐RADS 2–5 were noted in 111 women (8.2%); 1242 (91.5%) were negative; and 4 (0.3%) could not be assessed due to incomplete assessment (Table 2). Excluding these women, findings were assessed using a total of 1353 mammograms. Malignant mammographic features were seen in 1.3 % (*n* = 18) women, and 33.36% (*n* = 6) of these women were aged under 40 years. BI‐RADS categories for malignancy were reported in 16 (88.9%) of women aged 30 to 60 years. The lower risk Tabár Type I, II and III MPPs were associated with 94.4% (*n* = 17) of malignancies (Table 2).

**Table 1:** Mammographic findings as described by BI‐RADS category and age.

Mammographic findings (*N* = 1357)		Age in years						Age (years) Mean (SD)	95% Confidence interval
BI‐RADS	*N* (%)	<30	30–39	40–49	50–59	60–69	>70		
0	4 (0.3%)	0 (0%)	1 (25.0%)	1 (25.0%)	2 (50.0%)	0 (0%)	0 (0%)	43.0 (11.75)	24.31–61.69
1	1242 (91.5%)	163 (13.1%)	492 (39.6%)	449 (36.2%)	121 (9.7%)	15 (10.6%)	2 (0.2%)	39.1 (8.63)	38.58–39.54
2	67 (5.0%)	16 (23.9%)	18 (26.9%)	20 (29.9%)	7 (10.5%)	5 (7.5%)	0 (0%)	39.6 (11.52)	36.76–42.38
3	26 (1.9%)	4 (15.4%)	11 (42.3%)	7 (27.0%)	2 (7.7%)	1 (3.8%)	1 (3.8%)	38.9 (11.90)	34.08–43.69
4*	4 (0.3%)	1 (25%)	1 (25%)	0 (0%)	1 (25%)	1 (25%)	0 (0%)	43.75 (20.53)	11.08–76.42
5**	14 (1.0%)	1 (7.1%)	3 (21.4%)	4 (28.6%)	5 (35.7%)	1 (7.1%)	0 (0%)	47.4 (11.31)	40.83–53.89
6	0 (0)	0 (0%)	0 (0%)	0 (0%)	0 (0%)	0 (0%)	0 (0%)	43.0 (11.75)	24.31–61.69
	1357 (100%)	185 (13.6%)	526 (38.8%)	481 (35.5%)	138 (10.2%)	23 (1.7%)	3 (0.2%)		

The significance is that it highlights the high risk for cancer and malignant (cancer) mammographic features as distinct from the low risk for cancer and non‐malignant features.

* Suspicious for malignancy.

** Highly suspicious for malignancy.

**Table 2:** Mammographic findings as described by BI‐RADS category and Tabár Pattern Type I–V.

Mammographic findings (*N* = 1357)		Tabár pattern (type I–V)					Age (years) Mean (SD)	95% Confidence interval
BI‐RADS	*N* (%)	I	II	III	IV***	V***		
0	4 (0.3%)	3 (75%)	1 (25%)	0 (0%)	0 (0%)	0 (0%)	38.0 (7.85)	37.41–38.57
1	1242 (91.5%)	649 (52.3%)	375 (30.2%)	56 (4.5%)	84 (6.8%)	78 (6.3%)	42.9 (9.41)	42.00–43.83
2	67 (5.0%)	38 (56.7%)	14 (20.9%)	1 (1.5%)	10 (14.9%)	4 (6%)	41.5 (8.11)	39.35–43.62
3	26 (1.9%)	8 (30.8%)	13 (50%)	0 (0%)	4 (15.4%)	1 (3.8%)	38.6 (8.6)	36.87–40.32
4*	4 (0.3%)	4 (100%)	0 (0%)	0 (0%)	0 (0%)	0 (0%)	30.3 (7.69)	28.54–31.98
5**	14 (1.0%)	6 (42.8%)	6 (42.9%)	1 (7.1%)	0 (0%)	1 (7.1%)	38.0 (7.85)	37.41–38.57
6	0 (0)	0 (0%)	0 (0%)	0 (0%)	0 (0%)	0 (0%)		
	1357 (100%)	705 (52.1%)	408 (30.2%)	58 (4.3%)	98 (7.2%)	84 (6.2%)		

The significance is that it highlights the high risk for cancer and malignant (cancer) mammographic features as distinct from the low risk for cancer and non‐malignant features.

* Suspicious for malignancy.

** Highly suspicious for malignancy.

*** High‐risk Tabár pattern types.

**Figure 1:**
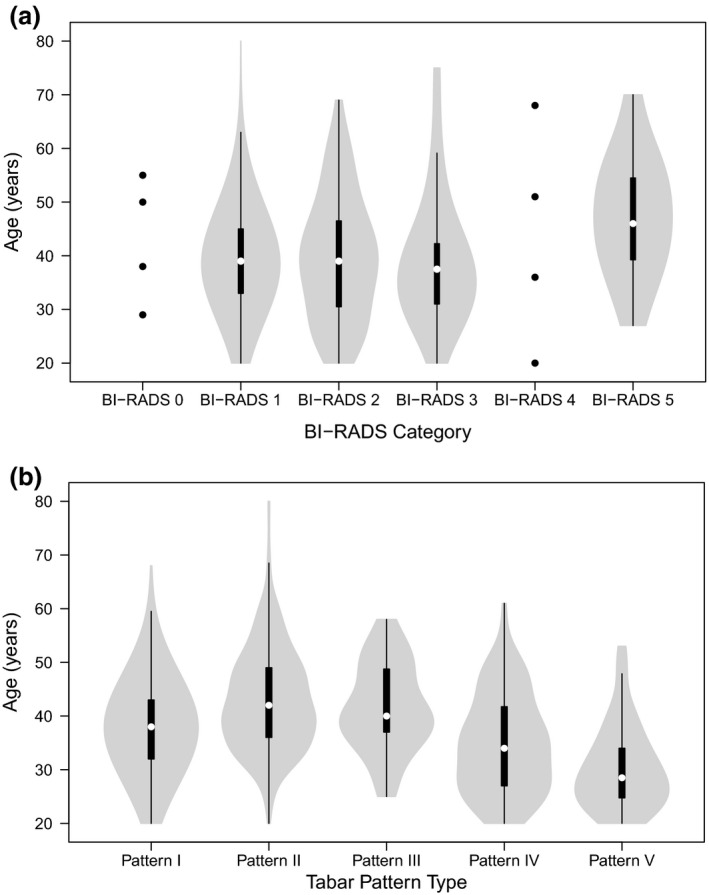
**(a)** and **(b):** Age versus BI‐RADS and Tabár Pattern distribution. Centre lines show the medians; box limits indicate the 25th and 75th percentiles as determined by R software; whiskers extend 1.5 times the interquartile range from the 25th and 75th percentiles, and outliers are represented by dots.

Violin plots have been used to illustrate the distribution of BI‐RADS categories and Tabár patterns by age (Fig. 1a,b). Analysis of age distributions using the Kruskal–Wallis test reported between group differences for both BI‐RADS (*P* = 0.341) and Tabár patterns (*P* < 0.0001).

For BI‐RADS, differences were seen between BI‐RADS 1 and 5 (*P* < 0.029) and BI‐RADS 3 and 5 (*P* < 0.032). All pair‐wise comparisons for Tabár pattern groups show statistical significant with the exception of Pattern II versus Pattern III; *P* values for other comparisons were *P* < 0.0001 except for Pattern I versus III (*P* = 0.047) and Pattern IV versus V (*P* = 0.002).

Collapsing Tabár groups I‐III as ‘low risk’ and Tabár ‘IV‐V’ as 'high risk' shows that the high‐risk group were younger (34.8 years, 33.43–36.11 [mean, 95% CI]) compared with those in the low‐risk group (39.9 years, 39.38–40.38, *P* < 0.001).

Linear correlations between variables were weak and not statistically significant; age and Tabár pattern *r* = 0.031, *P* = 0.0261; age and BI‐RADS *r* = 0.018, *P* = 0.517; Tabár pattern and BI‐RADS *r* = 0.020, *P* = 0.459 (*n* = 1357).

Almost half of the mammographic images reported with pathology affected the right breast 49.5% (*n* = 55) and the left breast 27% (*n* = 30), and bilateral pathology was seen in 23.4% (*n* = 26) of women (Table 3). Where mammographic and histopathological findings were reported in detail, there were 18 (16.2%) findings with malignant mammographic features (Table 3). Benign mammographic findings were reported in 93 (83.8%) of the breast images (Table 3). Reflective of reproductive age, cysts (72%) and fibroadenomas (76.6%) were most frequently diagnosed in women aged under 50 years (Table 4).

**Table 3:** Summary of mammographic findings as benign, malignant and left versus right breast pathology.

Breast Findings* (*N* = 111)		Benign Mammographic Features (N = 93) (83.8%)										Malignant Mammographic Features (N = 18) (16.2%)				
Location	N (%)	Masses (cysts/lumps)	Fibroadenoma	Hyperplasia	Calcification	Abscess	Fibromatosis	Lymphedema	Cutaneous lesions	Vascular disease	Adenosis (typical)***	Stellate masses	Increased densities with tissue distortion	Abnormal microcalcification (granular or casting types)	Invasive ductal carcinoma	Nipple and subcutaneous tissue retraction
		32 (34.4%)	30 (32.3%)	12 (12.9%)	4 (4.3%)	4 (4.3%)	4 (4.3%)	4 (4.3%)	2 (2.2%)	1 (1.1%)	0 (0.0%)	4 (22.2%)	4 (22.2%)	4 (22.2%)	4 (22.2%)	2 (11.1%)
Left	30 (27.0%)	12	10	2	0	2	0	2	0	0	0	0	1	0	1	0
Right	55 (49.5%)	16	12	4	3	2	0	2	2	0	0	3	3	3	3	2
Both**	26 (23.4%)	4	8	6	1	0	4	0	0	1	0	1	0	1	0	0
Total	111 (100%)	32	30	12	4	4	4	4	2	1	0	4	4	4	4	2

There was no typical (characteristic) adenosis diagnosed independently.

The significance is that it highlights the high risk for cancer and malignant (cancer) mammographic features as distinct from the low risk for cancer and non‐malignant features.

* Breast findings in this context refer to breast images reflecting both benign and malignant mammographic features.

** Breast findings of both benign and malignant mammographic features affecting both breasts.

*** Any breast images reported as having adenosis were diagnosed with other benign breast conditions such as masses and fibroadenomas.

**Table 4:** Summary of cysts and fibroadenomas versus age.

Age in years	Cysts *N* (%)	Fibroadenomas *N* (%)
<30	6 (18.8%)	10 (33.3%)
30–39	7 (21.9%)	6 (20.0%)
40–49	10 (31.3%)	7 (23.3%)
50–59	5 (15.6%)	3 (10%)
60–69	4 (12.5%)	4 (13.3%)
>70	0 (0.0%)	0 (0.0%)
Total	32 (100%)	30 (100%)

## Discussion

### Breast density

The mammographic density of the women of PNG has previously been reported by Pape et al. with no relationship between breast density profile and increased risk of breast cancer demonstrated.^21^ Pape et al. reported that density was not able to be identified as a causer to the high incidence of breast cancer in PNG.^21^ Reflective of these findings, results of the current study identify no statistically significant relationship between Tabár pattern and age and high‐risk BI‐RADS 4 and 5 findings. BI‐RADS 5 was reported in only one woman with a high‐density Tabár Type V MPP. This is in contrast to other studies which report the risk of breast cancer for women with high‐risk Tabár Type patterns as being increased when compared to the remaining patterns (OR 2.59) with prevalence reported as twice as high for these high‐risk patterns.^32^

It is noted that Tabár Type IV and V MPPs are typically only reported in a small number of women, approximately 10–12% and 5%, respectively, of an asymptomatic population.^33^ In the current study, Tabár pattern IV was reported in 7.2% of images and Tabár pattern V in 6% of images. Based on the PNG women able to access mammography, the results of this current study indicate that women of greater breast density, who are at higher risk of breast cancer, exhibit less breast cancer than those with lower density. The high‐risk group was noted to be younger (34.8, 33.43–36.11 [mean, 95%CI]) compared with those in the low‐risk group (39.9, 39.38–40.38, p < 0.001). This may be a reflection of the disproportion of women in each of the MPP categories and a limitation of this study.

### Mammographic abnormalities

In this study, there were nine women with calcification, BI‐RADS 2 (*n* = 3); BI‐RADS 3 (*n* = 2); and BI‐RADS 4 (*n* = 4). Pathology associated with calcification was determined to be benign (4.3%), malignant (22.2%) and indeterminate (61.4%). Nineteen women were reported to have mass lesions: BI‐RADS 2 (*n* = 10); BI‐RADS 3 (*n* = 7); and BI‐RADS 5 (*n* = 2). Pathology associated with mass lesions was determined to be benign (34.4%), malignant (22.2%) and indeterminate (55.5%).

In Australia, invasive ductal carcinoma is the most common breast cancer type for women aged 50–74 (78.4%).^34^ In this snapshot of PNG women, 22.2% (*n* = 4) of women with malignant mammographic features were diagnosed with invasive carcinoma. A lack of formalised pathological reporting in PNG has limited the value of these data. Women in PNG reported as having invasive cancers are advised to undergo breast surgery (mastectomy or lumpectomy) followed by radiotherapy and/or chemotherapy treatment which is currently not available in PNG.

The incidence of benign breast disease has been reported in an American study to increase with age from a rate of 22.6 per 1000 person‐years (2.3%) for women ages 25 to 29 to 35.6 per 1000 person‐year (3.6%) for women aged 40 to 44.^35^ The most common benign pathologic findings in the breast include cysts and fibroadenomas, and this was also found in this study with cysts accounting for 34.4% and fibroadenomas 32.3% of benign finding. Cysts and fibroadenomas can occur at any age, the peak incidence occurring during the second and third decades reflective of reproductive life;^5^ in this study, 72% of cysts and 76.6% of fibroadenomas were diagnosed in women younger than 50 years.

### Age

In Western countries, age is reported as the greatest risk factor for breast cancer. In Australia, more than three quarters of breast cancers occur in women over the age of 50.^34^ The results of this study highlight key differences between the needs of the women of PNG and those of Western nations. This study reflects the peak incidence of breast cancer in PNG previously reported by Halder et al. with 88.9% of women with breast cancer aged under 60 years, 66.7% under 50 years and 3.33% under 40 years.^2^ This supports previous reports that 80% of all breast cancer in PNG is premenopausal.^4^ The women of PNG seeking mammography screening are younger than that of most other countries who offer free screening services. This is a reflection of both the younger age of incidence and the reduced life expectancy for PNG women which was estimated to be 68 years in 2017.^35^ The majority of screening programs in the WHO European Region report screening target ages of 40–74, with only San Marino commencing target screening at under 40 years (35 years).^36^ Formalisation of the current screening program in PNG should consider establishing the baseline age of screening at 30 years with a target age group of between 30 and 60 years.

### Study Limitations

Due to the retrospective nature of this study and the limited record keeping of results in PNG, it was difficult to retrieve both biopsy reports and pathology results. Issues with record keeping were also encountered by Halder et al. and reflect a third world screening program in its infancy.^2^ The sample size is small and limited to women who were able to attend PIH for mammographic imaging and who had not already had surgery for breast cancer. Age distribution of this study in comparison with Western populations reflects the low life expectancy of the population of PNG and is as expected for a developing country.^36^ This is acknowledged as both a limitation of the study and a possible explanation of the result. It is also acknowledged that these results may not translate to the entire PNG screening population. This still remains an important study with this research being the first of its kind to report on relationships between these factors.

## Conclusion

This is the first study to report relationships between BI‐RADS category, age and MPP and report histopathological diagnosis of breast disease in the women of PNG since Halder et al. in 2001. Although limited by poor record keeping, this is an important study in that it fails to demonstrate correlation between BI‐RADS category, age and MPP. Importantly, there was no correlation demonstrated between high‐risk BI‐RADS categories 4 and 5 for breast malignancy with high‐risk Tabár Type IV and V breast density patterns. The small number of cancers in the high‐risk categories may limit the validity of this result.

Further research with greater scientific rigour and involving a larger population is required to validate the results of this study and to more accurately inform any policy development concerning a dedicated breast screening service in PNG. As BI‐RADS categories for malignancy were reported in 88.9% of women aged 30 to 60, this also suggests a need for any future screening program in PNG to be targeted at women much younger than that of Western countries.

## Conflict of Interest

The authors declare no conflict of interest.
